# Deep learning-based acceleration and denoising of 0.55T MRI for enhanced conspicuity of vestibular Schwannoma post contrast administration

**DOI:** 10.1007/s00234-025-03758-z

**Published:** 2025-09-19

**Authors:** Maximilian Hinsen, Armin Nagel, Rafael Heiss, Matthias May, Marco Wiesmueller, Claudius Mathy, Martin Zeilinger, Joachim Hornung, Sarina Mueller, Michael Uder, Markus Kopp

**Affiliations:** 1 Department of Radiology, Universitätsklinikum Erlangen, Germany; 2 Division of Medical Physics in Radiology, German Cancer Research Center, Heidelberg, Germany; 3 Krankenhaus Barmherzige Brüder, Regensburg, Germany; 4 Department of Otorhinolaryngology, Head and Neck Surgery, Universitätsklinikum Erlangen, Germany

**Keywords:** 0.55T MRI, Magnetic resonance imaging, Deep learning denoising of MRI, Head and neck imaging, Vestibular schwannoma, Low field MRI

## Abstract

**Background and purpose:**

Deep-learning (DL) based MRI denoising techniques promise improved image quality and shorter examination times. This advancement is particularly beneficial for 0.55T MRI, where the inherently lower signal-to-noise (SNR) ratio can compromise image quality. Sufficient SNR is crucial for the reliable detection of vestibular schwannoma (VS). The objective of this study is to evaluate the VS conspicuity and acquisition time (TA) of 0.55T MRI examinations with contrast agents using a DL-denoising algorithm.

**Materials and methods:**

From January 2024 to October 2024, we retrospectively included 30 patients with VS (9 women). We acquired a clinical reference protocol of the cerebellopontine angle containing a T1w fat-saturated (fs) axial (number of signal averages [NSA] 4) and a T1w Spectral Attenuated Inversion Recovery (SPAIR) coronal (NSA 2) sequence after contrast agent (CA) application without advanced DL-based denoising (w/o DL). We reconstructed the T1w fs CA sequence axial and the T1w SPAIR CA coronal with full DL-denoising mode without change of NSA, and secondly with 1 NSA for T1w fs CA axial and T1w SPAIR coronal (DL&1NSA). Each sequence was rated on a 5-point Likert scale (1: insufficient, 3: moderate, clinically sufficient; 5: perfect) for: overall image quality; VS conspicuity, and artifacts. Secondly, we analyzed the reliability of the size measurements. Two radiologists specializing in head and neck imaging performed the reading and measurements. The Wilcoxon Signed-Rank Test was used for non-parametric statistical comparison.

**Results:**

The DL&4NSA axial/coronal study sequence achieved the highest overall IQ (median 4.9). The image quality (IQ) for DL&1NSA was higher (M: 4.0) than for the reference sequence (w/o DL; median 4.0 versus 3.5, each p < 0.01). Similarly, the VS conspicuity was best for DL&4NSA (M: 4.9), decreased for DL&1NSA (M: 4.1), and was lower but still sufficient for w/o DL (M: 3.7, each p < 0.01). The TA for the axial and coronal post-contrast sequences was 8:59 minutes for DL&4NSA and w/o DL and decreased to 3:24 minutes with DL&1NSA.

**Conclusions:**

This study underlines that advanced DL-based denoising techniques can reduce the examination time by more than half while simultaneously improving image quality.

## Introduction

Patients with tinnitus, vertigo, and hearing loss have typical symptoms for a vestibular schwannoma (VS) [1]. However, these clinical symptoms lack specificity, and even deep learning–based evaluations of clinical and audiometric data do not substantially improve diagnostic accuracy; thus, further workup with MRI remains necessary [2, 3]. Therefore, contrast-enhanced MRI with almost 100% sensitivity and specificity remains essential for the detection of VS [3].

Despite significant technological advancements over the recent decades, access to MRI examinations remains unevenly distributed worldwide. One potential solution to this disparity is modern 0.55 T MRI, which offers reduced costs and operational requirements [4–6]. Moreover, MRI at lower field strengths has the intrinsic advantage of reduced susceptibility artifacts compared with higher field strengths [4, 7]. Consequently, 0.55 T MRI is particularly promising for imaging the cerebellopontine angle (CPA) where adjacent air-filled cavities in the mastoid cells and the petrous bone can exacerbate susceptibility artifacts at higher magnetic field strengths [8]. However, this physical advantage is counterbalanced by the generally lower signal-to-noise ratio (SNR) in 0.55 T MRI, which can compromise image quality [9]. A previous study demonstrated that while CPA imaging at 0.55 T is diagnostically usable, its image quality is lower compared to standard 1.5 T MRI [8].

Recent advances in artificial intelligence (AI), particularly in deep learning (DL), have led to new DL-based reconstruction methods that provide denoised image data [10–13]. These methods offer the potential to enhance image quality by interpolating k-space data using neural networks, which can result in improved image quality, reduced acquisition times, or both. Technical details regarding the algorithm design are available in the literature [12, 14]. This approach is particularly valuable for 0.55 T MRI, as it compensates for the lower MRI signal, which would otherwise require an increase in the number of signal averages (NSA) to enhance quality. Although initial studies have demonstrated the substantial potential of DL-based denoising methods, clinical data for patients with VS at 0.55 T remain scarce.

This retrospective study aims to compare the conspicuity and size of vestibular schwannomas (VS) between DL-based denoising reconstructions performed with high and minimum numbers of averages as well as conventional reconstruction methods without DL-based denoising.

## Materials and methods

### Ethical approval

This retrospective study was approved by the local ethics committee in accordance with the Declaration of Helsinki (Ethik-Kommission der Medizinischen Fakultät der Universität Erlangen-Nuernberg, Erlangen, Germany; No 24_79_BR).

### Study design

**Fig. 1:**
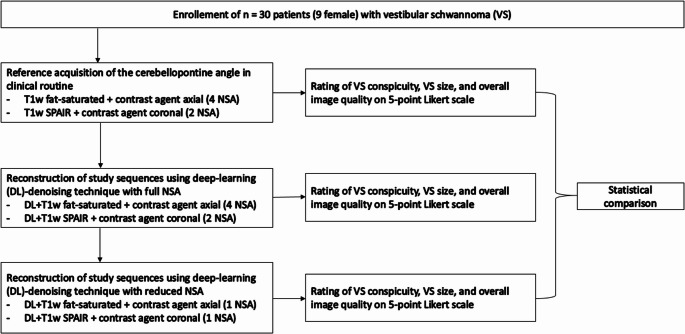
Illustration of study flow chart with illustration of reference acquisitions for the cerebellopontine angle and consecutive study sequences. Abbreviations: NSA – Number of Signal Averages; SPAIR - Spectral Attenuated Inversion Recovery; VS – Vestibular schwannoma

Between September 20, 2023, and October 22, 2024, an investigator-initiated exploratory trial was conducted at a single academic medical center. We enrolled thirty patients with vestibular schwannoma scheduled for 0.55 T MRI with contrast media administration. No examinations were repeated, and every patient gave consent to the MRI examination. Figure 1 illustrates the study flow chart.

### Imaging techniques

MRI examinations were performed at an 0.55 T MRI scanner (MAGNETOM Free.Max, Siemens Healthcare GmbH, Erlangen, Germany) using a 12- channel head/neck phased-array receiver coil. Reference transversal images of the CPA were acquired without advanced deep–learning based acceleration using a T1-weighted sequence with spectral fat-saturation (T1w fs). Acquisition parameters for the axial sequence included: TR/TER of 416/16 milliseconds (ms), an in-plane resolution of 0.4 x 0.4 mm^2^, 2.5 mm slice thickness, a number of excitations/averages (NSA) of 4, and a 256 x 256 matrix (acquisition time: 4:52 minutes). Coronal images were obtained using a T1-weighted reference sequence with Spectral Attenuated Inversion Recovery (SPAIR) fat-saturation (TR/TE 512/17 ms), with an in-plane resolution of 0.5 x 0.5 mm2, 2.5 mm slice thickness, a number of averages (NSA) of 2, and a 224 x 163 matrix (acquisition time: 4:07 minutes). All patients were set up in a head-first supine position with arms down.

Post-processing of the reference sequences was performed using an advanced DL-denoising technique (Deep resolve boost, Siemens Healthineers, Erlangen, Germany). The T1w fs axial study sequence was reconstructed using the DL-denoising technique without change of the NSA compared to reference standard (= 4 NSA) and with maximal possible NSA reduction to 1. The T1w SPAIR coronal study sequence was reconstructed using the DL-denoising technique, with the NSA maintained at the reference standard (= 2 NSA) and with maximal possible NSA reduction to 1. With 1 NSA and application of DL-denoising, the effective acquisition times were reduced to 1:18 minutes for the T1w fs axial and 2:06 minutes for the T1w SPAIR coronal.

The commercially available and regulatory approved deep learning-based reconstruction provided by the vendor was used. The model was trained on data acquired at 1.5T as well as 3T and was validated for 0.55T in the release process. The algorithm operates as a raw-data-to-image reconstruction technology, based on approximately 25,000 fully sampled images acquired at 1.5T and 3.0T. The DL-denoising technique is based on training data that includes various image contrasts, orientations, spatial resolutions, and anatomic regions. The vendor describes a retrospective down-sampling of imaging data during supervised training. The training consists of pairs of undersampled k-space raw data and fully sampled reference images at 1.5 T and 3 T [12, 14]. Additionally, Schlicht et al. and Schmidt et al. describe the feasibility of this DL-denoising technique for the knee and spine at 0.55T [15, 16]. In the present study, we did not perform fine-tuning of the model but followed the vendor-recommended ’used as-is’ calibration.

### Imaging evaluation

Pseudonymised MRI DICOM images were reviewed on a diagnostic monitor (EIZO RX660, Hakusan, Japan). The pseudonymized images were independently analyzed by two radiologists in a randomized order (reader one M.K. with 10 years of experience in cross-sectional imaging, reader two M.H. with 4 years of experience in cross-sectional imaging). A 4-week interval was maintained between the two reading sessions to avoid recall bias. Both readers were blinded to the patients’ medical history and the reconstruction parameters during the evaluation of image quality and size measurements. Both readers evaluated the image quality for each MRI sequences using the following categories on a five-point Likert scale (except category 2 and 4):

1. Overall image quality (1 = very low, 2 = low, 3 = medium, 4 = good, and 5 = very good).
2. Image artifacts in the relevant temporal region [inner ear and inner auditory canal (IAC); 1 = severe artifacts with insufficient IQ for clinical use, 2 = partly artifacts, which reduces IQ in the target structures, 3 = minor artifacts without reduced IQ, and 4 = optimal IQ without artifacts.
3. Conspicuity of VS (1: insufficient, 2: suboptimal, but partly diagnostic, 3: sufficient, but partly blurry conspicuity, 4: good, 5: optimal).
4. Decision of whether a VS was detectable or not (yes/no). Decision criteria were the signal intensity of an assumed VS compared to the adjacent neuronal and osseous tissue and the adjacent liquor-filled spaces. The decision was “yes” if a lesion revealed hyperintensity in close proximity to the vestibular nerve in the IAC or in the inner ear.

### Size measurement

A side-by-side comparison of two-dimensional size measurements was performed between each study sequence and the reference sequence. Volume measurements were not performed, as the DL-based denoising algorithm was not available for 3D, highly T2-weighted isotropic sequences of the CPA; thus, volumetric analysis was outside the scope of this sudy.

### Statistical analysis

For this exploratory study, we did not perform a formal a priori sample size calculation due to the lack of prior data on expected effect sizes for the investigated reconstruction methods at the studied field strength and body region. The results of this study will inform future power calculations for confirmatory research. Furthermore, the results of other research groups underline that a sample size of >10 is sufficient for MRI technical development studies [17, 18]:

Statistical analysis was performed using R (version 4.4.2, R Foundation for Statistical Computing, Vienna, Austria).

Adjusted p-values are reported with a p-value of <0.05 considered statistically significant. The Wilcoxon Signed-Rank Test for dependent samples was used to compare the subjective image quality between each MRI sequence. Data with a normal distribution are presented as means ± standard deviations, while LS scores, which were not normally distributed, are presented as medians and interquartile ranges (IQRs). Interrater agreement was assessed using weighted Cohen’s κ coefficients, calculated for the difference between the reference sequence and each study sequence. Kappa values ≥0.41 were interpreted as moderate, κ values ≥0.61 as substantial, and κ values ≥0.81 as almost perfect agreement according to Landis and Koch [19].

## Results

A total of 30 consecutive patients were included in the statistical analysis (9 female, 21 male). The mean patient age was 47 ± 16 years, and in 14 cases the VS was left sided.

### Overall image quality

**Fig. 2:**
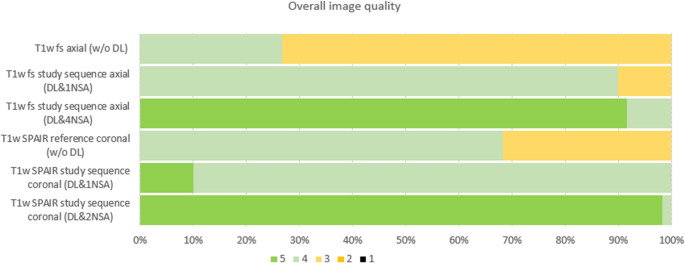
The bar chart displays the distribution of Likert scale ratings (1–5) for overall image quality across the study sequences including reference and study sequences with different noise suppression approaches (DL) and number of averages (NSA). Higher ratings (green) indicate better perceived quality, while lower ratings (yellow) reflect reduced image quality

**Fig. 3:**
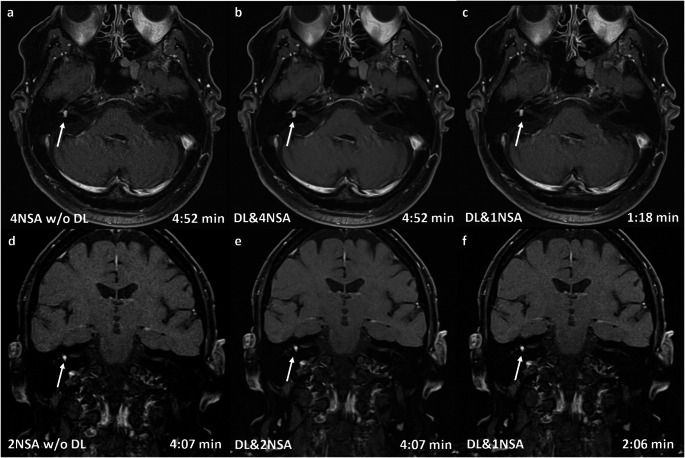
0.55 T cerebellopontine angle MRI of a 65-year-old male patient with a 6 × 5 × 4 mm intralabyrinthine vestibular schwannoma (VS) on the right side (arrow). All reconstructions—without (DL-) and with (DL+) a deep learning-based denoising algorithm, as well as with different numbers of averages (NSA)—in transversal (a–c) and coronal (d–f) orientations clearly show the contrast medium enhancing lesion on the right side. The overall image quality of the transversal sequences was rated highest for DL&4NSA (Likert scale: 5), followed by DL&1NSA (Likert scale: 4) and DL-4NSA (Likert scale: 3). The quality of the coronal images was rated highest for DL&2NSA (Likert scale: 5), the other reconstructions received an equal rating (Likert scale 4)

Overall image quality ratings were significantly higher for the DL-enhanced sequences compared to the reference sequences. Specifically, the reference T1w fs axial with CA (median 3, IQR 3-4) and the reference T1w SPAIR coronal sequence (median 3, IQR 3-4) were outperformed by the axial DL&4NSA sequence (median 5, IQR 5-5) and the coronal DL&2NSA study sequence (median 5, IQR 5-5; p < 0.001), respectively. In addition, the axial and coronal DL&1NSA sequences (both median 4, IQR 4–4) showed significantly higher ratings compared to their respective (non-accelerated) reference sequences (p < 0.001). Figure 2 illustrates the distribution of Likert scale ratings, and Figure 3 provides an example of the images acquired during the study.

### VS conspicuity

**Fig. 4:**
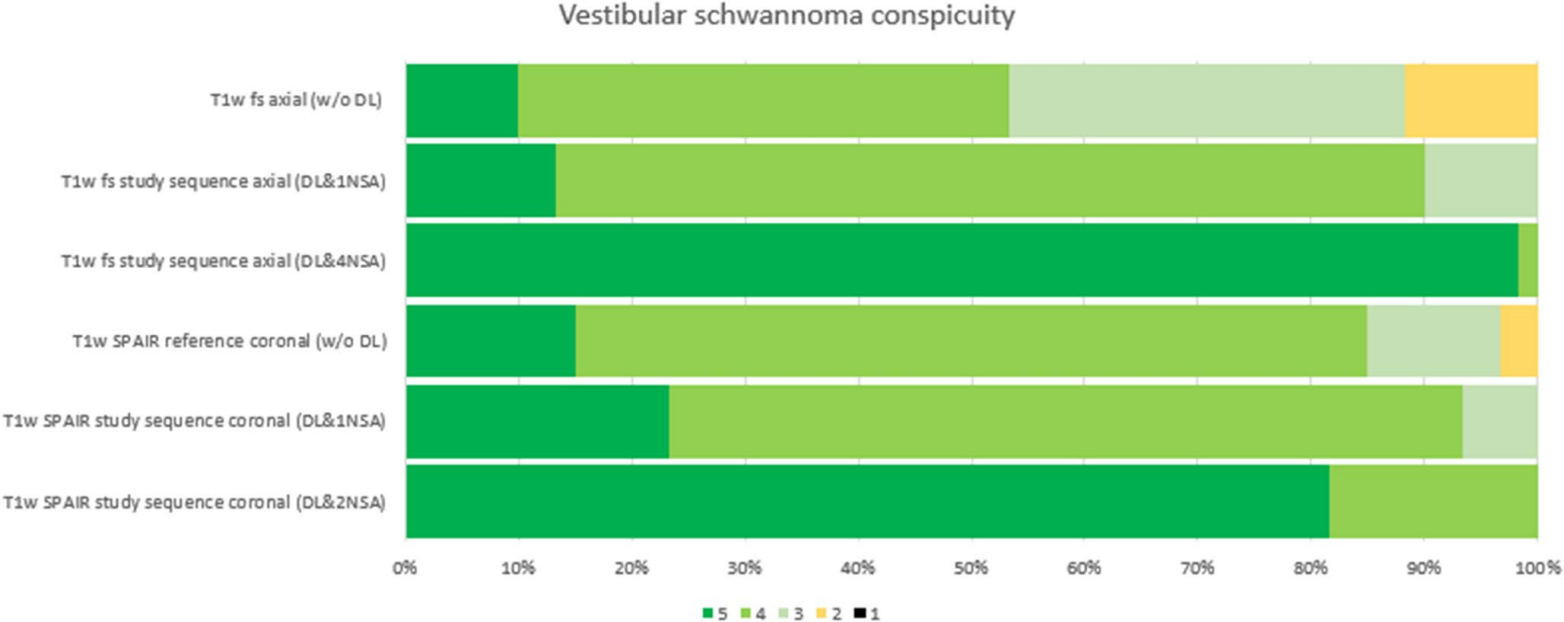
Bar chart of the Likert scale ratings from both readers regarding vestibular schwannoma conspicuity across the study sequences without (w/o DL) and with (DL) a deep learning-based denoising algorithm, as well as for different numbers of averages (NSA). The chart illustrates the higher conspicuity of vestibular schwannoma in sequences with denoising algorithm and, within this subgroup, for sequences with a higher number of averages

**Fig. 5:**
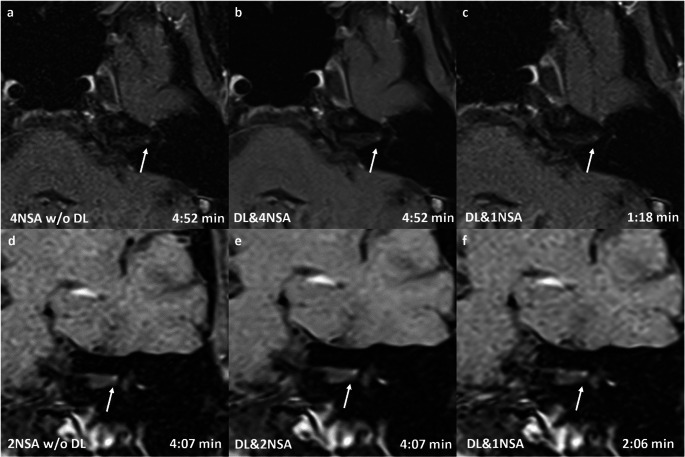
0.55 T MRI of 55-year-old female patient with punctual vestibularis schwannoma (VS) (maximum diameter 1mm) on the left site (arrow). All reconstructions without (w/o DL) and with (DL) deep learing based denosing algorithsm and with different number of averages (NSA) in transversal (a-c) and coronal (d-f) orientation) clearly show the enhancing lesion on the left site. The VS conspicuity of the transversal sequences was rated highest for DL&4NSA (Likert scale: 5), followed by DL&1NSA (Likert scale: 4) and DL w/o DL (Likert scale: 4). The quality of the coronal images was rated highest for DL&2NSA (Likert scale: 5), the other reconstructions received an equal rating (Likert scale 4)

The conspicuity of the VS was significantly higher for the DL-enhanced sequences. The reference T1w fs axial sequence (median 4, IQR 3-4) and the T1w SPAIR coronal sequence (median 4, IQR 4-4) were significantly inferior to the axial DL&4NSA (median 5, IQR 5-5) and coronal DL&2NSA sequence (median 5, IQR 5-5; each p < 0.01). A significant difference was also observed between the axial reference sequence and the axial DL&1NSA sequence (median 4, IQR 4-4; p < 0.001), whereas the VS conspicuity in the coronal plane did not differ significantly between the reference T1w SPAIR sequence and the coronal DL&1NSA sequence (median 4; IQR 4-4; p = 0.64). Figure 4 and 5 illustrate the Likert scale ratings and demonstrate the clear conspicuity of a small VS (maximum diameter 1 mm), respectively. 

Tables 1 and 2 show detailed kappa values and image quality results.

**Table 1:** Illustration of Kohens kappa calculation for the two expert readers regarding overall image quality, image artifacts and vestibular Schwannoma conspicuity. We provide 95% confidence intervals (CI) for each kappa value with lower an upper border, when applicable

	Overall image quality	95% CI	Image artifacts	95% CI	VS conspicuity	95% CI
T1w fs reference DL- axial	0.831 (CI95% (*p* < 0.001)	0.608-1.000	0.921 (*p* < 0.001)	0.762-1.000	0.851 (*p* < 0.001)	0.698-1.000
T1w fs study sequence axial (DL&4NSA)	0.783 (*p* < 0.001)	0.372-1.000	0.483 (*p* < 0.001)	0.030–0.935	–	–
T1w fs study sequence axial (DL&1NSA)	1 (*p* < 0.001)	1.000–1.000	0.923 (*p* < 0.001)	0.770-1.000	0.828 (*p* < 0.001)	0.600-1.000
T1w SPAIR reference DL- coronal	0.923 (*p* < 0.001)	0.776-1.000	0.793 (*p* < 0.001)	0.418-1.000	0.859 (*p* < 0.001)	0.669-1.000
T1w SPAIR study sequence coronal (DL&2NSA)	–	–	–	–	0,667 (*p* = 0.002)	0.320-1.000
T1w SPAIR study sequence coronal (DL&1NSA)	0.634 (*p* = 0,0019)	0.177-1.000	1 (*p* < 0.001)	1.000–1.000	0.859 (*p* < 0.001)	0.639-1.000

**Table 2:** Illustration of image quality (IQ) results for each sequence. We provide median values with interquartile ratio (IQR). The p value is referenced for the comparison between the reference routine sequence to each study sequence. The difference between T1w SPAIR coronal reference sequence and the T1w SPAIR study sequence with 1 NSA (number of signal averages) was non-significant (NS). The comparison of all other study sequence to the reference sequence showed significant (*p* < 0.001) differences regarding overall image quality and vestibular Schwannoma (VS) conspicuity

Image quality (IQ) evaluation	Overall IQ	*P* value	VS conspicuity	*P* value
T1w fs reference axial w/o DL	3, IQR 3–4	*p* < 0.001	4, IQR 3–4	each *p* < 0.001
T1w fs study sequence axial (DL&4NSA)	5, IQR 5–5		5, IQR 5–5	
T1w fs study sequence axial (DL&1NSA)	4, IQR 4–4		4, IQR 4–4	
T1w SPAIR reference coronal w/o DL	3, IQR 3–4	*p* < 0.001	4, IQR 4–4	both *p* < 0.001
T1w SPAIR study sequence coronal (DL&2NSA)	5, IQR 5–5		5, IQR 5–5	
T1w SPAIR study sequence coronal (DL&1NSA)	4; IQR 4–4		4; IQR 4–4	NS

### Overall image artifacts

**Fig. 6:**
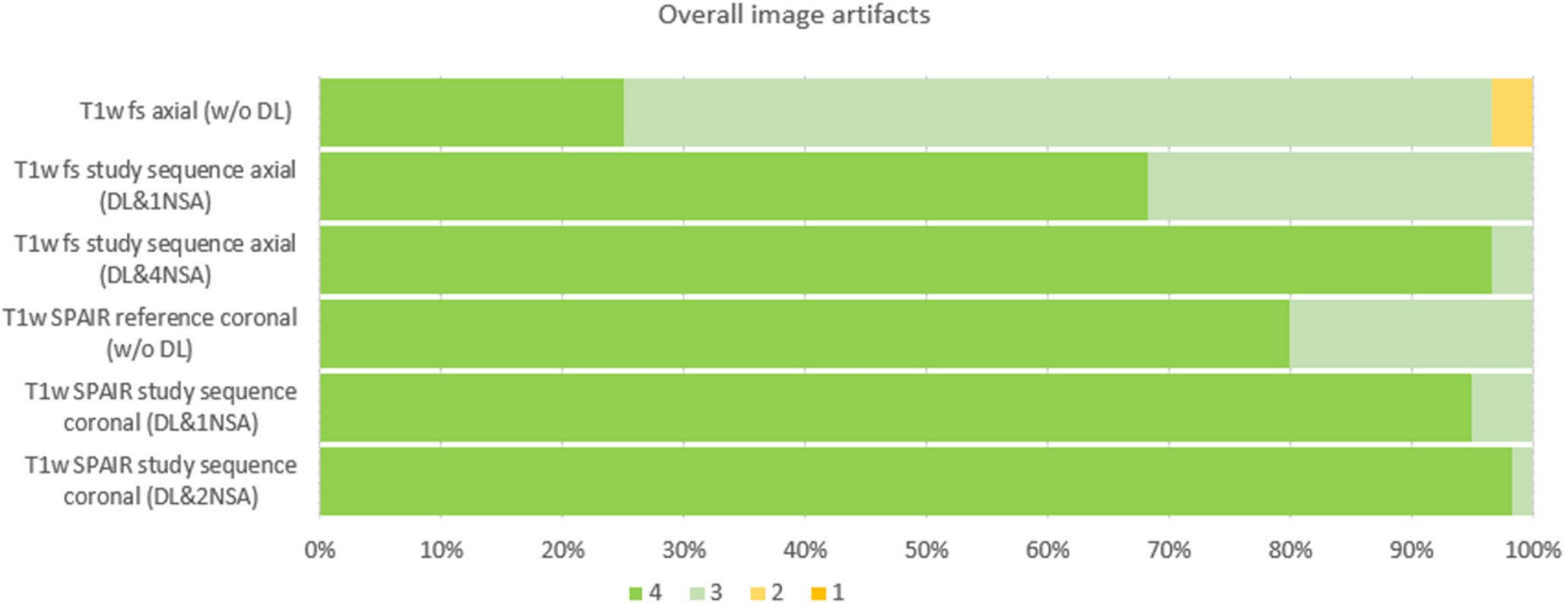
Bar chart of the Likert scale ratings from both readers regarding the overall image artifacts across reference and study sequences without (w/o DL) and with (DL) a deep learning-based denoising algorithm, as well as for different numbers of averages (NSA). The chart illustrates the lower artifacts (higher Likert scale) in sequences with denoising algorithm and, within this subgroup, for sequences with a higher number of averages. Likert scale 1 = severe artifacts, insufficient for clinical use, 2 = partly artifacts, which reduced image quality in the target structures, 3 = minor artifacts without reduced image quality, and 4 = optimal image quality without artifacts

Overall image artifacts were significantly lower in the DL-enhanced sequences, specifically the DL&4NSA (median 4, IQR 4–4) and DL&1NSA (median 4, IQR 4–4; p < 0.01). In the coronal sequences (median 4, IQR 4– 4), artifacts were also less common for both the DL&2NSA (p < 0.01) and DL&1NSA (p = 0.03) sequences. Figure 6 illustrates the distribution of Likert scale ratings for overall artifacts.

### Size Measurement

No statistically significant differences were found in the mean size measurements between the different sequences for all 90 measurements per reader (p>0.05). Details are illustrated in Table 3. Interrater agreement, as assessed by weighted Cohen’s κ, was 0.59, 0.74, and 0.78 for the axial sequences (w/o DL, DL&4NSA, and DL&1NSA, respectively) and 0.60, 0.79, and 0.71 for the coronal sequences (w/o DL, DL&2NSA, and DL&1NSA, respectively). No misclassification regarding the presence of VS occurred in the study or reference sequences.

**Table 3:** Illustration of mean size measurements and standard deviation for each sequence. The corresponding kappa values are given for the size measurement of each sequence

MRI sequence	Size measurement [mm]	kappa value
T1w fs reference axial w/o DL	62.3 ± 62	0.587
T1w fs study sequence (DL&4NSA) axial	64.9 ± 59.8	0.741
T1w fs study sequence (DL&1NSA) axial	62.9 ± 62.2	0.782
T1w SPAIR reference coronal w/o DL	55.2 ± 46.3	0.599
T1w SPAIR study sequence coronal (DL&2NSA)	56.5 ± 46.7	0.788
T1w SPAIR study sequence coronal (DL&1NSA)	56.7 ± 49.0	0.711

Abbreviations: *NSA* Number of signal averages; *SPAIR* Spectral attenuated inversion recovery; *VS* Vestibular Schwannoma

### Discussion

This study evaluates the capability of a DL-based denoising algorithm at 0.55T for improving the conspicuity of VS and high scan speed. By combining a DL-based based denoising technique with a cost-efficient 0.55T MRI system, we demonstrated that the acquisition time for contrast-enhanced study sequences can be reduced by 62% through a reduction in the NSA compared to the reference standard. In the axial plane, the DL&1NSA sequence provided improved image quality relative to the reference sequence (median 4 [4] vs 4 [3–4]), whereas in the coronal plane, the SPAIR DL&1NSA sequence achieved image quality equivalent to that of the reference (median 4 [4–4] vs. 4 [4–4]). When longer acquisition times are acceptable the axial/coronal DL&4NSA/2NSA sequences provide maximum conspicuity and overall image quality for VS. Furthermore, quantitative size measurements of the vestibular schwannoma were equivalent across both reference and DL-enhanced sequences, confirming that reducing the NSA does not compromise measurements accuracy in our study.

Deep learning-based denoising algorithms, particularly convolutional neural networks (CNNs), autoencoders, and generative adversarial networks (GANs), significantly enhance MRI image quality by effectively removing noise while preserving critical anatomical details. The algorithms used in this study uses raw-data-to-image deep learning reconstruction technology to reduce noise in the measured data.

This is particularly important in brain and head and neck imaging, where fine structural integrity is essential for accurate diagnosis [11, 13]. The reduction in acquisition time enabled by DL-based denoising could become a key instrument to overcome the uneven distribution of MRI availability. With reduced scan times, not only can more examinations be performed within the same time frame, but improved patient comfort could also result in less motion artifacts and makes it feasible to examine patients with severe medical conditions. The noise reduction is particularly promising in low-field MRI, which offers advantages such as lower costs and less demanding infrastructural requirements despite its inherently low SNR and longer examination times. However, to date, most clinical experience with DL-based denoising has been reported for musculoskeletal and brain imaging at conventional field strengths of 1.5 and 3.0 T [12, 20]. Herrmann et al. have reported notable improvements in image quality and scan time in musculoskeletal and body imaging using DL-based techniques (7-8). Additionally, ultra-fast MRI protocols — such as a 51-seconds EPI-FLAIR sequences accelerated with deep learning —have provided sufficient image quality for detecting inflammatory brain lesions >3mm [21]. To our knowledge, this study is the first to systematically evaluate the image quality improvement provided by DL-based denoising for head and neck imaging in low-field MRI.

This study has several limitations. First, only a rather small patient collective was available. Second, we did not evaluate the diagnostic performance in the detection of VS, as this was beyond the scope of the study. Third, although experienced radiologists with focus on head and neck imaging performed the image quality assessments, the semiquantitative nature of the ratings is prone to inter-reader variability. Despite a blinded reading of each of the six image groups, the DL-denoising algorithms is built to provide a low-noise image impression. We are aware this could also bias subjective semiquantitative ratings. Fourth, size measurement could not be validated against an external reference standard (e.g. surgical or histopathological data). Fifth, we were only able to conduct a retrospective study design as the positive rate for VS in the patient collective is rather small. Thus, we are aware of the inherent limitations concerning a potential selection bias. In a prospective study setting too many patients would have been included, who do not contribute to the scope of this study. Sixth, larger patient cohorts with a multi-center, prospective study approach are needed to verify our results on a broad clinical basis with AI-based MRI protocols from different imaging sites. Sevenths, we did not analyze all incremental steps from 4 NSA to 1 NSA using the deep learning-based denoising algorithm because we followed the approach to reduce the NSA to a minimum and compensate the increasing image noise by applying the present DL-denoising algorithm. Lastly, we are aware that patient workup for VS requires a highly T2w-weighted 3D-sequence, which, at the time of this study, was not available with DL-acceleration for the present 0.55T scanner and was not within the scope of this study. Deep learning–based acceleration of 3D MRI sequences is more challenging than 2D imaging due to higher computational and memory demands. Furthermore, interpolation techniques to reduce the overall size of image volume can cause information loss and also increased susceptibility due to undersampling artifacts should be considered [22, 23].

Advanced deep learning-based denoising algorithms are particularly promising in examinations with subtle changes and a low signal-to-noise ratio at 0.55 T. This study highlights the enormous potential of deep learning-based denoising for MRI assessments of vestibular schwannoma, demonstrating its capability to reduce examination time, improve image quality, or both.

## Data Availability

No datasets were generated or analysed during the current study.

## Abbreviations

CPA: Cerebellopontine angle
CA: Contrast agent
SPAIR: Spectral Attenuated Inversion Recovery
VS: Vestibular schwannoma
fs: Fat-saturation
